# Supplementary material for: Remarkable Recycling Process of ZnO Quantum Dots for Photodegradation of Reactive Yellow Dye and Solar Photocatalytic Treatment Process of Industrial Wastewater
Source: Nanomaterials (Basel). 2022 Jul 31;12(15):2642. doi: 10.3390/nano12152642 (PMC9370222; doi:10.3390/nano12152642)
Supplement: Supplementary file 1 [file nanomaterials-12-02642-s001.zip › nanomaterials-1834151-supplementary.pdf]

Table S1. COD values of real industrial wastewater using commercial nano zinc oxide, and ZQDs samples during photocatalysis by Sunlight.

Investigated Date	COD before treatment	COD Value (ppm)		
		Z1 (6.9 nm)	Z2 (8.3 nm)	Nano ZnO (25–30 nm)
3/2/2021	7020	790	978	1299
4/2/2021	6875	737	892	1152
7/2/2021	6378	658	808	925
8/2/2021	6544	702	842	954
9/2/2021	6101	620	775	887

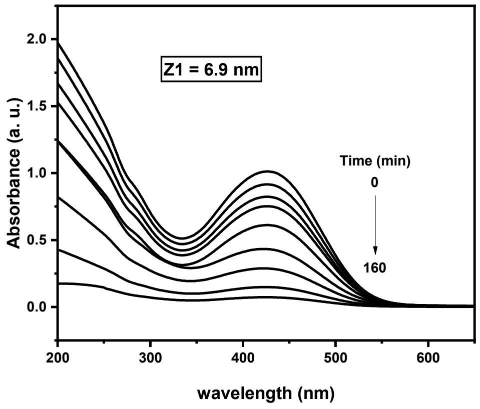

Figure S1. Absorption spectra for photodegradation process of Reactive Yellow Dye in presence of Z1 = 6.9 nm

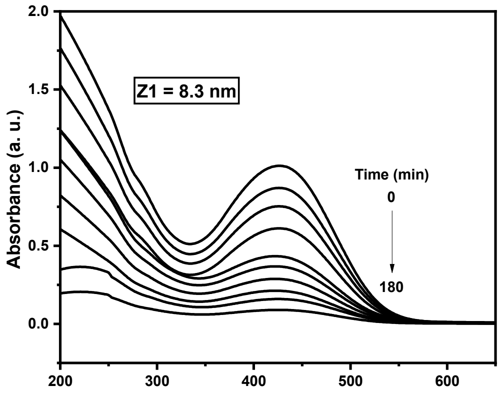

Figure S2. Absorption spectra for photodegradation process of Reactive Yellow Dye in presence of Z2 = 8.3 nm
